# Recent advances in managing brain metastasis

**DOI:** 10.12688/f1000research.15903.1

**Published:** 2018-11-09

**Authors:** Rupesh Kotecha, Vinai Gondi, Manmeet S Ahluwalia, Priscilla K Brastianos, Minesh P Mehta

**Affiliations:** 1 Department of Radiation Oncology, Miami Cancer Institute, Baptist Health South Florida, Miami, FL, USA; 2 Herbert Wertheim College of Medicine, Florida International University, Miami, FL, USA; 3 Northwestern Medicine Cancer Center Warrenville, Warrenville, IL, USA; 4 Northwestern University Feinberg School of Medicine, Chicago, IL, USA; 5 Rose Ella Burkhardt Brain Tumor and Neuro-Oncology Center, Taussig Cancer Institute, Cleveland Clinic, Cleveland, OH, USA; 6 Department of Hematology/Oncology, Taussig Cancer Institute, Cleveland Clinic, Cleveland, OH, USA; 7 Divisions of Hematology/Oncology and Neuro-Oncology, Massachusetts General Hospital, Boston, MA, USA; 8 Harvard Medical School, Boston, MA, USA

**Keywords:** brain metastasis, whole brain radiation therapy, stereotactic radiosurgery, neurocognition, targeted therapy, genomic

## Abstract

Brain metastases are the most common malignancy encountered in the central nervous system (CNS), with up to 30-40% of cancer patients developing brain metastases at some point during the course of their disease. The management of brain metastasis is rapidly evolving and the roles of local therapies such as whole-brain radiation therapy, stereotactic radiosurgery, and resection along with systemic therapies are in flux. An emphasis on the neurocognitive side effects associated with treatment has gained prominence. Novel molecular studies have demonstrated important evolutionary patterns underpinning the development of brain metastasis and leptomeningeal disease, which may be key to unlocking new therapeutic strategies. This article provides a framework for incorporating the results of recent randomized radiotherapy clinical trials into practice, expounds upon the emphasis on cognition being an important driver in therapeutic selection, describes the importance of CNS-penetrating systemic therapies, and provides an overview of the novel molecular insights that will likely set the stage for future developments in this field.

## Introduction

Brain metastases are the most common malignancy encountered in the central nervous system (CNS). Conventional therapeutic options have included resection, whole brain radiotherapy (WBRT), and stereotactic radiosurgery (SRS), with a very limited historical role for chemotherapy. However, targeted agents with blood-brain barrier (BBB)-penetrating capabilities, as well as immune checkpoint inhibitors (ICI) are expanding the role of systemic therapies in select subsets of patients, and current research focuses on identifying the best combinatorial approaches. WBRT has served as a component of treatment for several decades, but its role is rapidly evolving. It has proven beneficial in symptomatic patients for palliative relief, as primary treatment for brain metastases in patients who are expected to experience longer-term survival and in whom other treatments are not possible, as adjuvant therapy to lower recurrence rates after either resection or SRS, and as prophylactic treatment for systemic cancers that have a greater likelihood of intracranial spread. The cognitive toxicity of WBRT, however, cannot be ignored as the long-term sequelae can significantly impact quality of life; approaches that minimize this will be reviewed. SRS has been ascendant because of a lower cognitive dysfunction profile, and we shall review current clinical trials redefining the roles of these therapies. With the advent of targeted and immunological agents, the therapeutic landscape is shifting once again, and, as we shall demonstrate, novel molecular insights will most likely set the stage for revolutionary future advances.

## Overview of recent randomized radiotherapy clinical trials

Although WBRT is one of the standard treatment options for patients with brain metastasis, until recently, only one trial had compared the efficacy of WBRT vs. medical management. In 1971, the Eastern Cooperative Oncology Group (ECOG) reported on a study of 48 patients with brain metastases randomized to prednisone with or without WBRT ^1^. Clinical criteria were used to assess improvement in the patients’ status. WBRT prolonged both the duration of clinical remission (11 vs. 5 weeks, statistically significant, a relative improvement of 120%) and overall survival (median 14 vs. 10 weeks, relative improvement of 40%, but *P* = ns, underpowered to detect a survival benefit).

The value of WBRT was re-examined in the Medical Research Council (MRC) QUARTZ trial. In this study, 538 patients with non-small cell lung cancer (NSCLC) with brain metastasis unsuitable for surgery or SRS and in whom there was “uncertainty in the clinicians’ or patients’ minds about the potential benefit of WBRT” were randomized to dexamethasone and WBRT (20 Gy/5 fractions) versus dexamethasone and supportive care alone ^2^. This trial reported no difference in quality adjusted life days (46.4 with WBRT vs. 41.7 with supportive care; the measure of quality included dexamethasone-induced issues, and dexamethasone was used in both arms) - the primary endpoint of the study - and only a five day difference in median survival ^2^. What should practitioners take away from this trial? Quite simply, in patients with brain metastasis with a very short expected survival (similar to the poor outcomes observed in patients treated before the 1970s), hospice care and supportive management alone is appropriate.

The role of radiation therapy after surgery for patients with brain metastasis has been evaluated in randomized trials. The Patchell study, published 20 years ago, demonstrated that, in patients with a single brain metastasis, WBRT after surgery reduced the rate of surgical bed and distant brain relapse, and neurologic death ^3^. In the more recent MD Anderson Cancer Center (MDACC) study, patients who underwent resection of brain metastasis were randomized to observation or SRS. SRS improved the 12-month rate of local control (72% vs. 45%) and median time to local recurrence (not reached vs. 7.6 months) but SRS neither reduced the rate of distant brain failure nor did it improve overall survival ^4^. One important finding was the poor local control (<50% at 12-months) observed in lesions >2.5 cm, underscoring the need to improve outcomes through improved resection cavity delineation, dose-escalation, fractionated radiosurgery, or use of pre-op SRS ^5^. Although this trial supports the use of SRS after surgery, physicians need to caution patients about the significant risks of distant brain and/or leptomeningeal failure in the setting of focal therapy alone ^6^.

In the EORTC trial, after local therapy (SRS or surgery) patients were randomized to observation or WBRT. WBRT significantly reduced intracranial relapse (both local and distant) as well as neurologic death, but without prolonging overall survival ^7^. To explore SRS as an alternative to WBRT, the N107C trial compared these two modalities. Patients undergoing resection of a brain metastasis were randomized to WBRT or SRS to the resection cavity with SRS allowed to other intact metastases. The primary endpoint, cognitive deterioration-free survival, was judged to be a single standard deviation reduction in any single cognitive domain. Although there was a two week benefit in cognitive preservation in the SRS arm compared to the WBRT arm (approximately the time needed to deliver WBRT), more striking was the six-month rate of cognitive deterioration in both arms (85% for WBRT and 52% for SRS, a rather high rate of cognitive decline, not previously well-described as a consequence of SRS) ^8^. The rate of surgical bed control was higher in patients who received WBRT (78% vs. 57%) as was overall brain control (70% vs. 32%). Therefore, SRS is a reasonable option for patients after surgery, but patients should be appropriately counseled about the high rates of neurocognitive decline after SRS (especially because SRS is frequently presented as modality with negligible cognitive deficits), as well as the enhanced risks of local and distant brain failure, without a survival advantage over any modality.

Two randomized trials had previously evaluated the role of WBRT in addition to SRS for patients with limited brain metastases. The benefit of WBRT in reducing local relapse (27–33% without WBRT vs. 11–19% with WBRT) as well as distant brain failure (48–64% without WBRT vs. 27–42% with WBRT) was observed in both ^7, 9, 10^. The N0574 study re-examined this question by randomizing patients with 1–3 brain metastases to SRS with or without WBRT. Consistent with previous studies, this trial also demonstrated benefits of WBRT in reducing local failure (27 vs. 10%) and distant brain failure (30% vs. 8%) ^11^. However, there was improvement in the rate of cognitive deterioration (64% vs. 92% at 3 months) as well as improvement in quality-of-life in patients treated with SRS alone. Therefore, primary SRS is a reasonable option for patients with limited brain metastases, albeit with only modest impact on neurocognition and quality-of-life. In practice, we should carefully select the patients who receive each of these modalities and identifying patients at high risk for neurologic death or intracranial failure may help further inform these decisions ^12^.

The aforementioned trials have clarified the role of WBRT in patients with poor expected survival, validated the need for adjuvant radiotherapy after surgery for brain metastasis, demonstrated the advantages and limitations of SRS after surgery in comparison to WBRT, and supported the role of single modality focal therapy in patients with limited brain metastases, as long as the substantially enhanced risk of intracranial failure is acceptable to the patient.

## Cognition as a key driver in therapeutic selection

Surveys of brain metastasis patients and oncology nurses reveal cognition as an important factor in patient preferences for treatment ^13^. Several randomized trials have demonstrated cognitive decline following WBRT, with cognitive function measured using a validated, multi-dimensional battery of tests assessing episodic memory (HVLT-R), executive function (TMT Part B, COWA), processing speed (TMT Part A), and fine motor control (Grooved Pegboard) ^8, 10, 11^. These studies have observed an improvement in the rate of 3-standard deviation decline of cognitive testing from baseline to 3-6 months post-treatment from 35–52% in WBRT vs. 6–24% after SRS ^8, 11^. Interestingly, a significant proportion of the cognitive difference between WBRT and SRS seems to selectively involve decline in HVLT-R, implying a differential sensitivity of episodic memory to WBRT. 

However, demonstration of these cognitive effects has important limitations. Since most studies use a time-to-event analysis, these trials are limited in their capacity to assess for later recovery, following initial decline in cognitive function. However, since many brain metastasis patients on these trials did not live beyond six months, the relevance of such longer-term follow-up can be questioned. In addition, while trials have demonstrated cognitive and quality-of-life effects following WBRT, one study observed decline in episodic memory in addition to self-reported cognitive complaints; however, minimal correlation was seen between decline in tested versus patient-reported cognitive function ^14^. Such discordance between objectively measured and patient-reported cognitive function has been seen in other cognitive disorders such as Alzheimer’s dementia, and highlights the need to objectively measure cognition with performance-based neuropsychological tests along with patient-reported outcomes ^15^. Alternatively, it could also provide the almost heretical conclusion that patients care less about cognitive test scores and focus more on daily life activity issues that these tests do not measure, a very sobering thought!

In a small minority of patients (less than 5%), WBRT can be associated with debilitating dementia, which represents the more severe end of the spectrum of cognitive decline reported in clinical trials ^16^. This severe form of radiation-induced toxicity can manifest as progressive dementia, gait ataxia and urinary incontinence – especially in patients treated with hypofractionated schedules (>3.5 Gy/fraction) ^16^.

Over the past decade, there have been significant advances in the development and testing of pharmacologic and technologic approaches for reducing cognitive decline following WBRT. Memantine is a non-competitive, low-affinity, open-channel antagonist of the N-methyl-D-aspartate (NMDA) receptor that blocks pathologically excessive stimulation of NMDA receptors. In pre-clinical models, memantine has been shown to be neuro-protective and in two placebo-controlled trials it proved to be an effective treatment for vascular dementia ^17– 20^. RTOG 0614 randomized 554 patients with brain metastases receiving WBRT to either memantine or placebo ^21^. Although this study did not meet its pre-defined endpoint, multiple other endpoints were either clinically or statistically significant. For example, patients who were randomized to the memantine arm experienced a longer time to cognitive decline as well as a reduced risk for cognitive failure following treatment (54% vs. 65%, *P* = 0.01). Furthermore, memantine was neuroprotective in multiple cognitive domains including executive function, processing speed, and delayed recognition.

Recently, conformal avoidance of the neural stem-cell bearing subgranular zone of the hippocampal dentate gyrus using intensity-modulated radiotherapy (IMRT) during WBRT has shown significant promise in preventing cognitive toxicity from WBRT. The production of new neurons from mitotically active neural stem cells found in the subgranular zone of the hippocampal dentate gyrus is key to the creation of new memories ^22^. The results of preclinical studies show that damage to these neural stem cells from even low doses of radiation underpin radiotherapy-induced cognitive toxicity. Additional clinical studies have established a dose-response relationship between the dose received by the hippocampus and risk of post-radiotherapy decline in episodic memory ^23^. Modern hippocampal avoidance (HA-WBRT) IMRT techniques have been developed to conformally avoid the hippocampal dentate gyrus while still covering the at-risk brain parenchyma ^14^. RTOG 0933, a single-arm phase II trial, demonstrated that HA-WBRT was associated with highly promising preservation of memory and quality-of-life, as compared to pre-specified historical controls. Specifically, the primary endpoint on this trial, the mean relative decline in HVLT-R delayed recall score from baseline to four months was only 7%, significantly better in comparison to the 30% decline in the historical control ( *P* <0.001). To validate these findings, NRG CC001 (ClinicalTrials.gov identifier NCT02360215), a phase III, 518-patient trial of WBRT with memantine (M) with or without hippocampal avoidance for patients with brain metastases with the primary outcome of time to neurocognitive failure (NCF) was completed and presented at the ASTRO Annual Meeting in 2018. Time to NCF failure was significantly longer in favor of HA-WBRT+M. The NCF failure rates following WBRT+M vs. HA-WBRT+M were 63.0% vs. 53.7% at four months, and 69.1% vs. 58.0% at six months ( *P* = 0.012).

NRG CC003 (ClinicalTrials.gov identifier NCT02635009) is an ongoing randomized phase II/III trial of prophylactic cranial irradiation with or without hippocampal avoidance for small cell lung cancer with the primary outcomes of intracranial relapse rate and six-month deterioration in episodic memory; the randomized phase II component has completed accrual.

Prior studies comparing cognitive outcomes following SRS and WBRT have not included these neuroprotective strategies that may reduce cognitive decline following WBRT. Thus, the optimal selection of SRS versus WBRT in the modern era of brain metastasis management remains an area of important investigation.

## The advent of systemic therapies

Traditional cytotoxic chemotherapy had a limited role in the management of brain metastases due to the presence of the BBB and such therapies were associated with low response rates ^24, 25^. With the advent of targeted therapies and immunotherapy, the role of medical therapy is experiencing a resurgence. Targeted therapies have mostly been evaluated in subsets of patients with lung cancer, breast cancer, and melanoma. The use of first generation tyrosine kinase inhibitors (TKIs) that act on *EGFR*-mutant NSCLC brain metastases, such as erlotinib and gefitinib, are associated with response rates of 50–80%, and overall survival of 12–24 months ^26– 28^. Trials with third generation TKIs such as osimertinib are associated with response rates of 55–70% with more durable responses ^29, 30^. In the AURA -3 study, osimertinib yielded response rates of 70% ^30^. Newer agents that target EGFR include AZD-3759 ^31, 32^ and avitinib (targets the EGFR T790M resistant mutation) ^33^. In NSCLC brain metastases with *ALK* translocations, the first generation inhibitor crizotinib resulted in response rates of 18% ^34^. Newer generation drugs such as alectinib ^35^, ceritinib ^36^, and brigatinib ^37, 38^ (a combined ALK and EGFR inhibitor) with better ability to cross the BBB ^39^, have resulted in responses rates of 45–78%.

In breast cancer patients with brain metastases, most targeted agents have been evaluated in the HER2-positive setting ^40^. Lapatinib, a small molecule TKI inhibitor of HER2, has limited activity as a single agent, and has been combined with capecitabine ^41– 43^. In phase II studies, the lapatinib-capecitabine combination results in response rates of 66% in radiotherapy-naive patients and 20% in radiation refractory patients ^41– 43^. Neratinib has demonstrated limited efficacy with responses rates of 8% in HER2-positive brain metastases ^44^; however, response rates improve to 49% with capecitabine ^45^. Other drugs being examined in this patient population include tucatinib and tesevatinib ^46, 47^.

Of patients with brain metastases from melanoma, 40–50% harbor BRAF mutations, and the use of the BRAF inhibitor vemurafenib is associated with 18–20% response rates and dabrafenib yields 30–40% response rates ^48, 49^. Similar to breast cancer patients, higher response rates are seen in patients that are radiation naive. The dabrafenib/trematinib combination is associated with response rates of 55% ^50^. The duration of response with dabrafenib and trematinib is approximately six months.

Immunotherapies represent an exciting area of research in brain metastases. Drugs that target immune surface proteins CTLA4 (ipilimumab) and programmed cell death protein 1 (PD1) (pembrolizumab and nivolumab) have been developed and evaluated in patients with lung cancer and melanoma brain metastases ^51^. A phase II trial of ipilimumab demonstrated disease control rates of 25% in those who were not on steroids and 10% in patients on steroids ^52^. Phase II trials of pembrolizumab showed response rates of 22% in melanoma and 33% in NSCLC ^53^. The combination of ipilimumab and nivolumab is associated with response rates of 45–57% (in some studies, stable disease is included in this measure) in patients with melanoma brain metastases ^54– 56^. Most of the initial trials evaluated these ICIs or receptor tyrosine kinase inhibitors (RTKIs) alone in brain metastases. A number of retrospective studies have shown that these agents can safely be used with SRS and have shown improved clinical benefit compared to those treated with SRS alone, or ICIs/RTKIs alone ^57^. A number of ongoing trials are therefore evaluating these agents in combination with WBRT or SRS.

With increasing CNS penetration and intracranial efficacy with systemic therapies, a current dilemma in clinical practice is the use of upfront SRS at time of brain metastasis diagnosis or delayed SRS in patients who fail systemic therapies. Retrospective data using a strategy of systemic therapy with delayed SRS are mixed, with some series demonstrating no difference in survival ^58, 59^, while others show a detriment to patient outcome with upfront systemic therapy alone ^60^. Given the limited data in this setting, a randomized study is clearly needed to determine the optimal sequencing of available therapies.

## Novel molecular insights set the stage for the future

Recent advances in genomic technologies and analytic tools have enhanced our understanding of the genetics of brain metastases. Unanswered questions have included whether brain metastases are genetically heterogeneous compared to their primary tumors, and whether differential clinical responses can be explained by such genetic heterogeneity.

A massively parallel sequencing study of one matched brain metastasis and a primary breast cancer showed two *de novo* mutations and a deletion in the metastasis and not in the primary tumor ^61^. In a comprehensive genomic study of 104 matched brain metastases and primary tumors across multiple histologies and a variety of treatment regimens, investigators mapped out the phylogenetic relationship between brain metastases and primary tumors ^62^. An evolutionary pattern of ‘branched’ or divergent evolution was ubiquitously observed, meaning the primary tumor and brain metastasis shared a common ancestor, yet there was significant divergent evolution in each site. As a result of this branched evolution pattern, in more than 50% of cases, clinically actionable alterations were present in the brain metastasis, and not detected in the primary tumor. This implies that genomic characterization of the primary tumor alone to identify therapeutic targets may miss potentially clinically significant alterations in the brain metastasis. Notably, when multiple regional and anatomically distinct brain metastases were analyzed, the majority of clinically actionable alterations were shared among the intracranial sites, suggesting genomic homogeneity within the brain itself. Furthermore, extracranial metastases (as opposed to the primary tumor) displayed divergent evolution and were not a genetic surrogate for clinically actionable alterations in the brain metastases. These data suggest that genetic heterogeneity, at least in part, contributes to divergent therapeutic responses within the same patient. This study also demonstrated that alterations in the CDK, PI3K/AKT and HER2/EGFR pathways were common in brain metastases, suggesting that targeting these pathways should be considered. Other investigations have confirmed an enrichment of the PI3K/PTEN pathway in brain metastases when compared to extracranial sites in melanoma ^63^, squamous cell lung cancers ^64^ and breast cancer ^65^. FGFR amplifications are also more enriched in brain metastases from lung adenocarcinomas compared to primary tumors, and also represent a potential therapeutic target for brain metastases patients ^66^. 

Clinical trials should be conducted to answer the question of whether targeting the alterations specific to the brain metastasis will lead to improved clinical outcomes. Large-scale molecular studies of brain metastases across multiple histologic tumor types are needed to identify additional therapeutic targets. Nevertheless, molecular analysis of brain metastasis tissue, if available as part of clinical care, should be considered to identify potential targeted therapies.
